# Medial Rectus Muscle Injuries After Functional Endoscopic Sinus Surgery: A Case Study

**DOI:** 10.7759/cureus.64212

**Published:** 2024-07-10

**Authors:** Mariya A Alhashim, Amal I Alhemidan

**Affiliations:** 1 Ophthalmology, Prince Sultan Military Medical City, Riyadh, SAU; 2 Ophthalmology/Pediatric Ophthalmology and Strabismus, Prince Sultan Military Medical City, Riyadh, SAU

**Keywords:** retrobulbar hemorrhage, diplopia, medial rectus muscle injury, ophthalmic complications, functional endoscopic sinus surgery (fess)

## Abstract

Functional endoscopic sinus surgery (FESS) is the preferred method for managing obstructive sinus disorders. However, its proximity to the orbit poses a risk of orbital complications.

This study presents a case of a 61-year-old female who underwent FESS for chronic rhinosinusitis with nasal polyps and subsequently experienced a serious ophthalmic complication including retrobulbar hemorrhage and medial rectus muscle hematoma, leading to adduction deficit and diplopia.

The patient's condition was evaluated through clinical assessment and imaging studies, to address the extent and nature of the injury to the medial rectus muscle. Management strategies included surgical exploration and resection along with botulinum toxin injection to the lateral rectus muscle in the affected eye done six months after observation and regular ophthalmic examination to ensure the stability of the angle of deviation.

This case highlights the importance of proper preoperative assessment and personalized treatment plans to manage the complications associated with FESS and optimize patient outcomes.

## Introduction

Functional endoscopic sinus surgery (FESS) stands as the procedure of choice for addressing obstructive sinus disorders. Due to the proximity of the paranasal sinuses to the orbit, there is a risk of orbital content injury during sinus surgery. Ophthalmic complications may be categorized as follows: minor (grade I) injuries involve damage to the lamina papyracea, major (grade II) injuries involve the lacrimal duct, and serious (grade III) complications encompass retroorbital hemorrhage, optic nerve injury leading to vision reduction or blindness, and extraocular muscle damage [1,2]. The medial rectus (MR) muscle is the extraocular muscle most frequently injured during FESS because of its proximity to the thin medial orbital wall [3].

We documented a case involving a serious ophthalmic complication characterized by retrobulbar hemorrhage and MR muscle hematoma, resulting in adduction deficit and diplopia.

## Case presentation

A 61-year-old female, with a history of bronchial asthma, obesity (BMI 39), and hypothyroidism, was diagnosed with stable right-sided middle and posterior cranial fossae meningiomas. On August 7, 2023, she underwent FESS for chronic rhinosinusitis with nasal polyps. The procedure included polypectomy, anterior ethmoidectomy, opening of the basal lamina, clearance of the posterior ethmoid, and bilateral turbinoplasty. During frontal sinusotomy in the left nasal cavity, there was a susception of anterior ethmoid artery injury, but there was no bleeding or signs of orbital hemorrhage intraoperative, even after half an hour of intraoperative observation.

**Figure 1:**
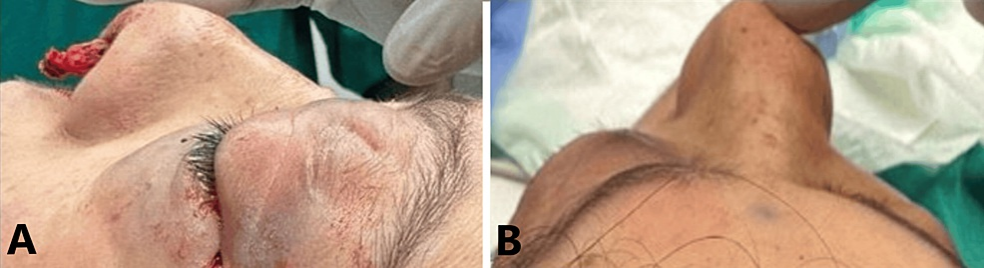
Signs of retrobulbar hemorrhage shown in A and B. A showing left eyelid swelling, ecchymosis, and inability to open the eye. B showing left eye proptosis.

Shortly after being shifted to the recovery area, the patient experienced severe eye pain, tense eyelids, and inability to open the eye. She was diagnosed with acute retrobulbar hemorrhage (Figure 1). The patient was promptly taken back to the operating theatre, where a lateral canthotomy and cantholysis were done under local anesthesia within less than 15 minutes. She received intravenous mannitol 20% 1 g/kg and intravenous dexamethasone 40 mg TID.

**Figure 2:**
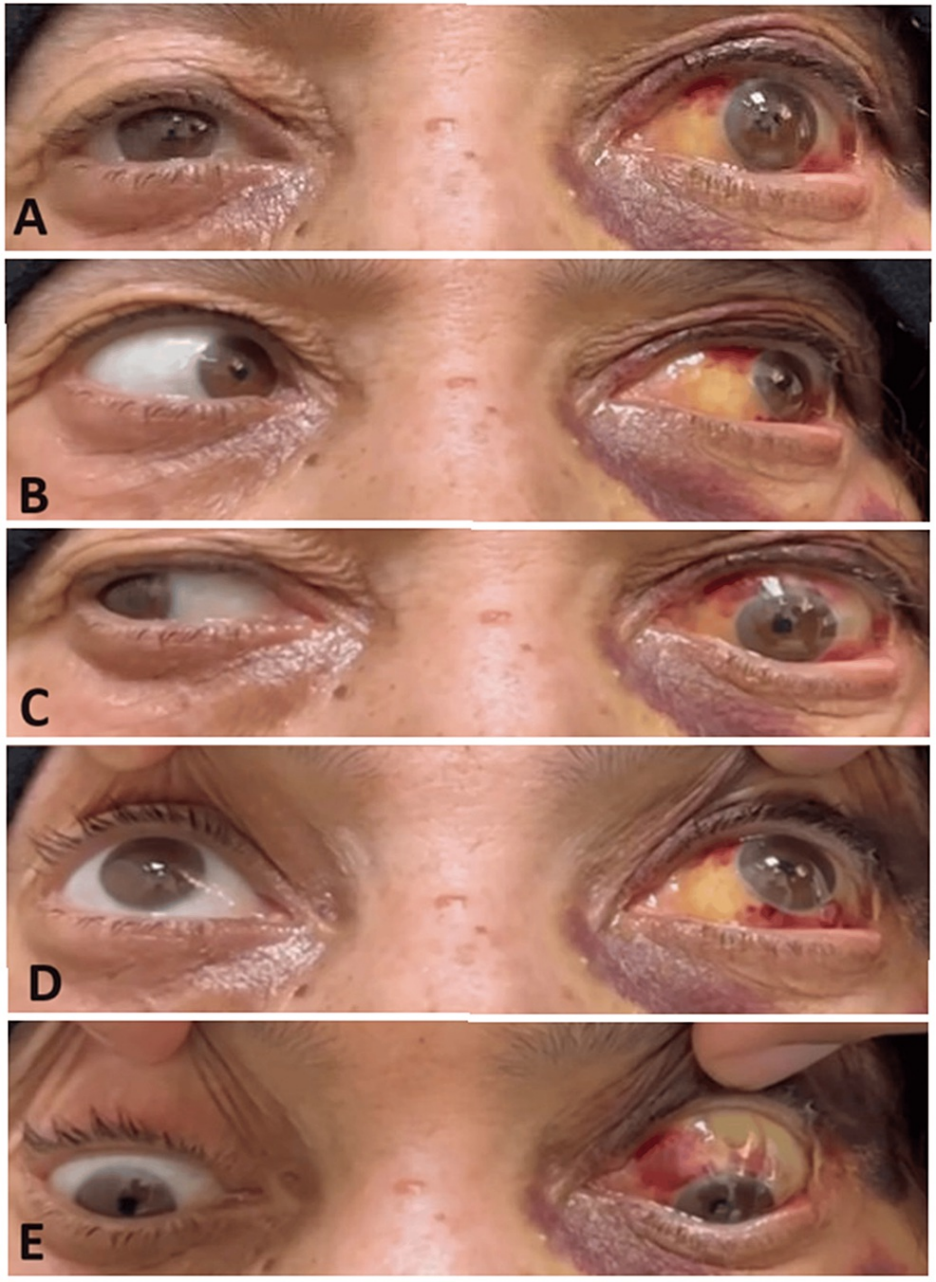
Day 1 post-lateral canthotomy and cantholysis of the left eye. A showing exotropia in primary gaze. B showing full abduction in the left eye. C showing -4 limitation of adduction in the left eye. D showing full supraduction in both eyes. E showing full infraduction in both eyes.

On the same day, the patient complained of double vision. An ophthalmic examination was performed and showed a visual acuity of 20/30 in both eyes and intraocular pressure of 12 mmHg in the right eye and 21 mmHg in the left eye using Reichert Tono-Pen AVIA. Full extraocular muscle movement was documented in the right eye; however, there was limited adduction (-4) with 50 dioptres of exotropia in the left eye (Figure 2). Pupil examination showed round regular reactive pupils without afferent pupillary defect in both eyes. Slit lamp examination of the right eye was unremarkable, while the left eye exhibited severe swelling of the superior and inferior lids, diffuse subconjunctival hemorrhage, clear lens, healthy optic disc and macula, flat retina, and no signs of central retinal artery occlusion.

Two weeks later, the patient was still complaining of double vision and was covering that eye with an eye pad all the time to be able to function normally. Suturing of the cantholysis was performed.

Four weeks postoperative, the patient underwent a peribulbar injection of 40 mg of methylprednisolone to reduce the postoperative edema and swelling under sedation. Follow-up examinations revealed a limitation of adduction (-3) and 40 prism dioptres of exotropia in the left eye and horizontal binocular diplopia.

**Figure 3:**
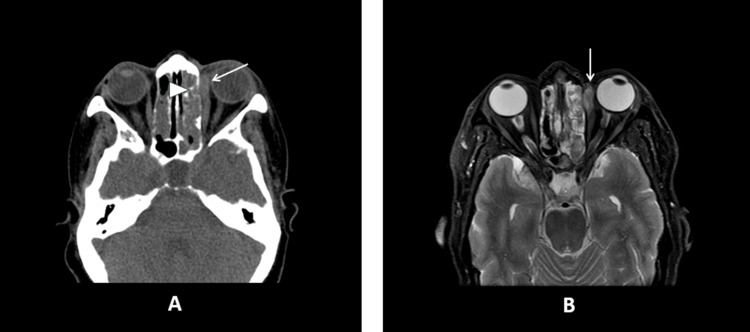
First orbital CT and MRI with contrast. (A) Axial CT image showing heterogeneous high to intermediate attenuation of the left medial rectus muscle (straight arrows). There is questionable dehiscence of lamina papyracea (arrowhead). (B) Axial T2-weighted MR image showing heterogeneous high signal intensity of the anterior left medial rectus indicating intramuscular hematoma (straight arrows).

During the first orbital CT on August 14 (one week postoperatively as the patient was not medically stable enough for shifting to the radiology department) which was comparable to the MRI with contrast, the left orbital showed a well-defined intramuscular hematoma involving the anterior aspect of the MR muscle of the left eye. The myotendinous junction of the left MR muscle could not be visualized (Figure 3).

There is no evidence of ruptured MR. However, there is a hematoma in the medial orbit involving the MR muscle and the adjacent tissues. The muscle is inseparable from the surroundings, and the muscle belly to the apex is clearly visible; however, the MR muscle looks thinned behind its insertion.

During the follow-up period, the patient underwent a series of orbital imaging.

**Figure 4:**
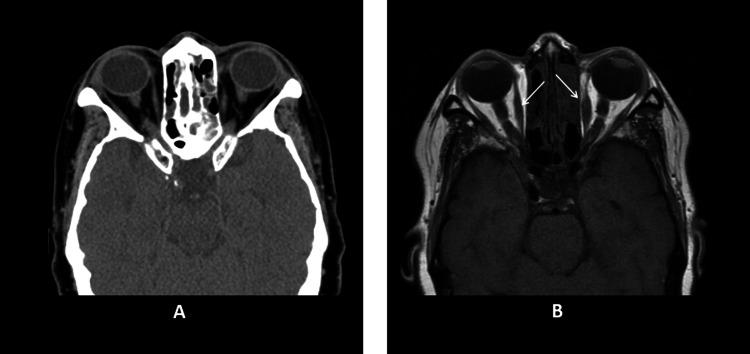
Serial imaging CT and MRI with contrast. Follow-up axial CT image and T2-weighted MR image showing interval resolution of the previously seen hematoma in the left medial rectus muscle with residual fatty stranding, abnormal shape of the muscle, and thinned tendon with irregular margins (A, B). Compare the abnormal left to the normal right (straight arrows) (B).

MRI with contrast in the following three months was performed and showed interval evolution of the left intra-orbital hematoma which has markedly decreased in size with residual patchy areas of abnormal signal (Figure 4).

**Figure 5:**
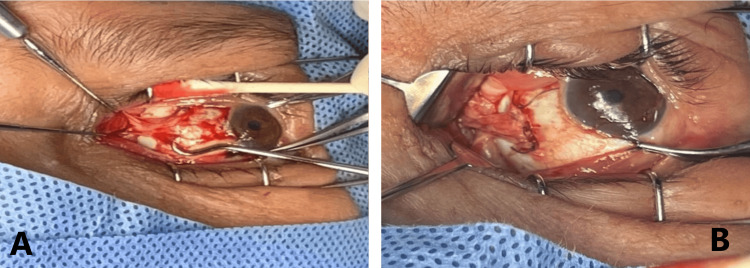
Intraoperative photos of the left medial rectus. A showing the thin atrophic medial rectus muscle before resection. B showing the thin atrophic medial rectus muscle after resection was performed.

After six months of follow-up, as the angle of deviation was the same and there were limitation of adduction and binocular double vision, it was decided to explore the MR muscle. Consequently, the patient underwent the planned procedure under general anesthesia; the MR muscle was extremely thin with atrophy of most muscle fibers. MR resection of 6 mm was performed using the standard technique, along with a lateral rectus botulinum injection of 7 units, where 1 ml of balanced salt solution (BSS) was used to dilute 100 U of botulinum toxin (Allergan) under sterile condition following which 0.7 ml was withdrawn into 27-gauge needle on a syringe and injected to the muscle under direct visualization (Figure 5).

**Figure 6:**
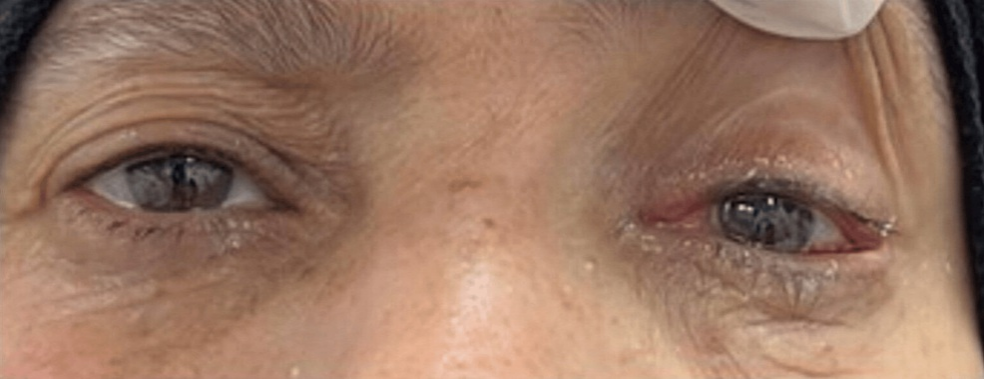
Post-op alignment of the eye (day 1): the patient exhibited orthophoria with immediate disappearance of diplopia.

On the first day postoperatively, the patient exhibited orthophoria with immediate disappearance of diplopia (Figure 6). Two weeks after surgery and six months follow-up visit post-surgery show orthophoria, no diplopia, and no relapse of symptoms. 

## Discussion

In recent years, FESS has become the preferred surgical preference for addressing obstructive sinus disorders that do not respond to medical treatment. While generally considered safe, FESS can lead to either minor or major complications [4].

The most common ophthalmic complication is orbital hemorrhage, with additional reported complications in the orbit such as optic nerve injury, extraocular muscle damage, and injuries to the nasolacrimal drainage system [5]. 

The incidence of orbital hemorrhage post-FESS varies depending on the study period and the targeted population. Ramakrishnan et al. reported orbital complications in 0.07% of patients [6], while Dalziel et al. found that orbital hematomas occurred in 0-4% of their study population [7]. However, a study conducted at King Saud University Medical City in Riyadh by Alharbi and his colleagues found that two out of 1395 patients (0.14%) had orbital hematomas [8].

Recent studies showed that the MR is the most injured extraocular muscle [2]. Bhatti et al. reported nine cases of extraocular muscle injury during FESS; the primary entry point into the orbit was predominantly the lower medial orbital wall (seven out of nine cases), with subsequent injury to the MR muscle occurring in four out of nine cases. Injuries to the inferior rectus were observed in two out of nine cases, while injury to the superior oblique muscles was noted in one out of nine cases [9]. The largest study to date in FESS was about MR muscle-related injuries [10]. Fontes et al. reported that three patients presented with total rupture of the MR muscle after FESS and were surgically treated within 3-7 days [11].

Diplopia and limited extraocular motility after FESS may arise from either muscle entrapment within a wall defect or damage to the vascular or neural supply of the extraocular muscle which is the most likely explanation in our case as the muscle tendon was intact but severely atrophic and nonfunctioning resulting in adduction deficit and diplopia that persisted up to six months postoperative [2].

Huang et al. analyzed 30 cases of MR injury following FESS in the largest reported case series. They identified four distinct patterns of MR muscle injury: pattern I described as a complete transection of the MR muscle, characterized by a large-angle exotropia, significant adduction deficit, and relatively preserved abduction; pattern II, partial transection or severe contusion of the MR, with or without entrapment, presenting as moderate- to large-angle exotropia with both adduction and abduction deficits; pattern III, grossly intact MR with marked muscle and soft tissue entrapment, characterized by a small-angle esotropia and a significant abduction deficit; and lasts pattern IV, muscle contusion without entrapment, resulting in varying degrees of ocular misalignment [10].

Managing patients with extraocular muscle injury resulting from FESS poses a significant challenge. Effective treatment hinges on understanding the injury's mechanism, location, and severity. It's crucial to differentiate between restrictive and paretic mechanisms through forced duction testing, as the latter often leads to temporary ocular motility dysfunction that may resolve spontaneously. Forced duction testing provides valuable insights for surgical planning and prognosis assessment [12]. The forced duction test of our patient showed no MR restriction with mild restriction of the lateral rectus which was insignificant indicating the paretic nature of the injury that did not resolve.

In cases categorized as pattern I according to Huang et al.'s classification, involving a complete transection of the MR, immediate orbital exploration is warranted to reconnect the severed muscle ends. Pattern II injuries can be managed similarly to pattern I. Pattern III injuries are managed akin to medial wall fracture repair, along with releasing the entrapped MR. For patients like ours classified as pattern IV, a conservative approach over 3-6 months, and sometimes up to 12 months, is warranted for those experiencing contusive, neural, or vascular damage to the extraocular muscles. During this period, the antagonist rectus muscle may be injected with botulinum toxin to prevent contracture.

Thacker et al. suggest that including multi-positional or dynamic MRI scanning in the standard protocol is advisable for managing these cases. Dynamic MRI allows the visualization of muscle contractility, offering functional evaluation of the extraocular muscles. Also, they recommend attempting retrieval and reattachment only if muscle contractility is evident on multi-positional MRI scans or if the patient presents within three months of the acute phase, given the potential for nerve damage recovery [13].

Thorough preoperative evaluation, including imaging to identify pre-existing anatomical differences and assessing the course and position of the anterior ethmoid artery which is the involved artery in these cases, can reduce the occurrence of postoperative complications. Anatomical anomalies such as pre-existing lamina papyracea dehiscence, changes in anatomy from previous sinus surgeries or trauma, and limited visualization due to significant intraoperative bleeding increase the likelihood of orbital and extraocular muscle complications during sinus surgery [14].

## Conclusions

Extraocular muscles are at risk of inadvertent damage during FESS. The timing and approach to treatment hinge on the extent and type of injury, as well as the involvement of multiple extraocular muscles. Effective treatment plans rely on clinical examinations and CT or MRI findings. Surgical approaches should be tailored to individual cases for optimal outcomes.

## References

[1] Dunya IM, Salman SD, Shore JW (1996). Ophthalmic complications of endoscopic ethmoid surgery and their management. Am J Otolaryngol.

[2] Rene C, Rose GE, Lenthall R, Moseley I (2001). Major orbital complications of endoscopic sinus surgery. Br J Ophthalmol.

[3] Mukherjee B, Priyadarshini O, Ramasubramanian S, Agarkar S (2015). Iatrogenic injury to medial rectus after endoscopic sinus surgery. Indian J Otolaryngol Head Neck Surg.

[4] May M, Levine HL, Mester SJ, Schaitkin B (1994). Complications of endoscopic sinus surgery: analysis of 2108 patients--incidence and prevention. Laryngoscope.

[5] Eitzen JP, Elsas FJ (1991). Strabismus following endoscopic intranasal sinus surgery. J Pediatr Ophthalmol Strabismus.

[6] Ramakrishnan VR, Kingdom TT, Nayak JV, Hwang PH, Orlandi RR (2012). Nationwide incidence of major complications in endoscopic sinus surgery. Int Forum Allergy Rhinol.

[7] Dalziel K, Stein K, Round A, Garside R, Royle P (2006). Endoscopic sinus surgery for the excision of nasal polyps: a systematic review of safety and effectiveness. Am J Rhinol.

[8] Alharbi A, Alhussain F, Alyamani A (2023). Complications of endoscopic sinus surgery for chronic rhinosinusitis in a tertiary care teaching hospital in Saudi Arabia. Saudi Med J.

[9] Bhatti MT, Schmalfuss IM, Mancuso AA (2005). Orbital complications of functional endoscopic sinus surgery: MR and CT findings. Clin Radiol.

[10] Huang CM, Meyer DR, Patrinely JR (2003). Medial rectus muscle injuries associated with functional endoscopic sinus surgery: characterization and management. Ophthalmic Plast Reconstr Surg.

[11] Fontes ÉB, Felippu AW, Felippu AW (2020). Surgical reconstruction technique of medial rectus muscle after endoscopic sinus surgery iatrogenic rupture - report of three cases*. Rhinology Online.

[12] Underdahl JP, Demer JL, Goldberg RL, Rosenbaum AL (2001). Orbital wall approach with preoperative orbital imaging for identification and retrieval of lost or transected extraocular muscles. J AAPOS.

[13] Thacker NM, Velez FG, Demer JL, Rosenbaum AL (2004). Strabismic complications following endoscopic sinus surgery: diagnosis and surgical management. J AAPOS.

[14] Driben JS, Bolger WE, Robles HA, Cable B, Zinreich SJ (1998). The reliability of computerized tomographic detection of the Onodi (sphenoethmoid) cell. Am J Rhinol.

